# CAR-T Versus Non-CAR-T Bridging Strategies Before Allogeneic Hematopoietic Stem Cell Transplantation in Relapsed/Refractory B-Cell Acute Lymphoblastic Leukemia: A Systematic Review

**DOI:** 10.7759/cureus.115393

**Published:** 2026-08-29

**Authors:** Omar Halloumi, Sanae Sabbar, Laila Lahlou, Salma Fares

**Affiliations:** 1 Department of Clinical Hematology and Cellular Therapy, Mohammed VI University Hospital Center, Agadir, MAR; 2 Department of Hematology, Hôpital Universitaire de Bruxelles (H.U.B.), Institut Jules Bordet, Bruxelles, BEL; 3 Department of Hematology, Faculty of Medicine and Pharmacy of Agadir, Ibn Zohr University, Agadir, MAR; 4 Laboratory of Epidemiology and Clinical Research, Faculty of Medicine and Pharmacy of Agadir, Ibn Zohr University, Agadir, MAR

**Keywords:** allogeneic hematopoietic stem cell transplantation, bridging therapy, car-t cells, measurable residual disease, relapsed/refractory b-cell acute lymphoblastic leukemia, systematic review

## Abstract

Relapsed or refractory B-cell acute lymphoblastic leukemia (R/R B-ALL) remains a major therapeutic challenge despite recent advances in immunotherapy. Anti-CD19 chimeric antigen receptor T-cell (CAR-T) therapy has emerged as a promising bridging strategy to allogeneic hematopoietic stem cell transplantation (allo-HSCT), although its impact on post-transplant outcomes compared with non-CAR-T bridging strategies remains uncertain. This systematic review compared CAR-T-based with non-CAR-T bridging strategies before allo-HSCT in patients with R/R B-ALL or persistent/recurrent measurable residual disease (MRD) during first complete remission (CR1).

PubMed/MEDLINE and Scopus were searched from database inception to August 18, 2026. Eligible comparative studies reported post-transplant outcomes according to the pre-transplant bridging strategy. Two reviewers independently screened the studies, extracted data, and assessed methodological quality using the appropriate Joanna Briggs Institute (JBI) critical appraisal tools. Given the substantial clinical and methodological heterogeneity, a narrative synthesis was performed rather than a meta-analysis.

Of 1,957 records identified, 10 comparative reports met the eligibility criteria. Potential patient-level overlap could not be excluded between two reports from the same institution, and all included studies were conducted at single-center institutions in China. CAR-T-based bridging produced deep pre-transplant remissions, but no consistent advantage over non-CAR-T strategies in achieving MRD negativity was observed. One study reported a significantly lower cumulative incidence of relapse (CIR) and higher disease-free survival (DFS) after CAR-T bridging (18.9% vs. 42.2%, p = 0.02; 82.5% vs. 53.7%, p = 0.01), with CAR-T remaining independently associated with a reduced risk of relapse in multivariable analysis. A significant unadjusted overall survival (OS) advantage favoring CAR-T was observed in one small pediatric cohort (84.6% vs. 40.0%, p = 0.008), although potential overlap with another included cohort limits the independent interpretation of this finding. In the largest cohort, CAR-T bridging was independently associated with improved OS in multivariable analysis (hazard ratio = 0.365, 95% CI 0.154-0.857, p = 0.025), despite a nonsignificant unadjusted OS comparison and significantly lower leukemia-free survival (LFS). Across the remaining studies, OS did not differ significantly between bridging strategies, including in two studies comparing CAR-T with blinatumomab.

Non-relapse mortality (NRM), hematopoietic engraftment, graft-versus-host disease (GVHD), infectious complications, and endothelial toxicity showed no consistent direction of effect across studies, although delayed platelet recovery and increased viral reactivation emerged as potential safety signals in specific cohorts, particularly following dual-target CD19/CD22 CAR-T therapy. Overall, CAR-T-based bridging before allo-HSCT may improve disease control in selected patients with R/R B-ALL, but the expanded evidence base does not demonstrate a consistent survival or safety advantage over non-CAR-T bridging strategies. The choice of bridging strategy should therefore be individualized according to MRD status, disease biology, CAR-T construct, and transplant eligibility. Prospective multicenter comparative studies are needed to define the optimal sequencing of CAR-T therapy, alternative immunotherapies, and allo-HSCT.

## Introduction and background

B-cell acute lymphoblastic leukemia (B-ALL) is an aggressive hematologic malignancy driven by the uncontrolled proliferation of immature B-cell precursors within the bone marrow. It encompasses a broad spectrum of genetic abnormalities, including BCR::ABL1 fusion, KMT2A rearrangements, hyperdiploidy, and Philadelphia chromosome-like (Ph-like) alterations, which increasingly guide prognostic assessment and treatment decisions [1,2]. Contemporary pediatric protocols now achieve five-year overall survival (OS) rates exceeding 90%, whereas outcomes in adults remain considerably less favorable and depend heavily on age and disease risk [1,3]. The Global Burden of Disease 2021 analysis highlights the substantial worldwide burden of ALL, particularly in resource-limited settings [4]. Nationwide epidemiological data remain scarce in Morocco, although the Greater Casablanca Cancer Registry confirms a measurable burden of lymphoid leukemias in the country [5].

Despite substantial progress in frontline therapy, relapse and primary refractory disease remain major causes of treatment failure in B-ALL, and durable disease control after conventional salvage chemotherapy remains uncommon. Targeted immunotherapies, particularly blinatumomab and inotuzumab ozogamicin, have broadened the therapeutic options available for relapsed or refractory B-ALL. In the phase III TOWER trial, Kantarjian et al. [6] showed that blinatumomab improved median OS compared with standard chemotherapy (7.7 vs. 4.0 months) and achieved a higher rate of complete remission with full, partial, or incomplete hematologic recovery (44% vs. 25%). In the INO-VATE trial, Kantarjian et al. [7] reported that inotuzumab ozogamicin achieved a substantially higher rate of complete remission or complete remission with incomplete hematologic recovery than standard chemotherapy (80.7% vs. 29.4%). Real-world experience with the sequential use of these two agents has subsequently illustrated their potential to achieve disease control and facilitate progression to allogeneic hematopoietic stem cell transplantation (allo-HSCT) [8].

More recently, anti-CD19 chimeric antigen receptor T-cell (CAR-T) therapy has emerged as another major therapeutic advance in relapsed/refractory (R/R) B-ALL, producing high complete remission rates frequently accompanied by measurable residual disease (MRD) negativity, even in heavily pretreated patients. However, durable disease control is not guaranteed. Several mechanisms of relapse after CD19-directed CAR-T therapy have been described, including CD19 antigen loss or downregulation, persistence of residual leukemic clones, and progressive loss of CAR-T-cell persistence or function [9]. For this reason, allo-HSCT after CAR-T-induced remission has increasingly been used as a consolidation strategy in selected high-risk patients, with long-term clinical experience supporting the relevance of this sequential approach [10].

However, whether CAR-T provides a superior pre-transplant bridging strategy compared with non-CAR-T approaches remains uncertain. The available comparative evidence is predominantly non-randomized and heterogeneous with respect to patient age, disease status, CAR-T construct, comparator treatment, transplant eligibility, and transplant procedures [9,10].

Against this background, we conducted a systematic review comparing CAR-T-based with non-CAR-T bridging strategies before allo-HSCT in patients with R/R B-ALL or persistent/recurrent MRD during first complete remission (CR1). The primary objective was to evaluate post-transplant survival and disease control; secondary objectives included relapse, non-relapse mortality (NRM), hematopoietic recovery, graft-versus-host disease (GVHD), infectious complications, and other transplant-related toxicities.

## Review

Methods

Study Design

This systematic review was designed and reported in accordance with the Preferred Reporting Items for Systematic Reviews and Meta-Analyses (PRISMA) 2020 statement by Page et al. [11]. It was not prospectively registered in PROSPERO or another systematic review registry.

Research Question and PICO Framework

The review examined whether, among patients with B-ALL undergoing allo-HSCT, CAR-T-based bridging was associated with different post-transplant outcomes compared with non-CAR-T bridging strategies. The research question was structured according to the PICO (Population, Intervention, Comparator, Outcomes) framework (Table 1).

**Table 1:** Research Question Structured According to the PICO Framework Allo-HSCT, allogeneic hematopoietic stem cell transplantation; B-ALL, B-cell acute lymphoblastic leukemia; CAR-T, chimeric antigen receptor T-cell; CIR, cumulative incidence of relapse; CMV, cytomegalovirus; CR1, first complete remission; DFS, disease-free survival; EBV, Epstein-Barr virus; EFS, event-free survival; GRFS, graft-versus-host disease-free/relapse-free survival; GVHD, graft-versus-host disease; LFS, leukemia-free survival; MRD, measurable residual disease; NRM, non-relapse mortality; OS, overall survival; PFS, progression-free survival; Ph, Philadelphia chromosome; R/R, relapsed/refractory; TA-TMA, transplant-associated thrombotic microangiopathy; TKI, tyrosine kinase inhibitor; VOD, veno-occlusive disease

Component	Definition
P - Population	Adult and pediatric patients with relapsed/refractory (R/R) B-ALL, or CR1 with persistent/recurrent MRD, candidates for allo-HSCT. Ph-positive and Ph-negative disease included.
I - Intervention	CD19-targeted CAR-T therapy ± CD22 (dual-target), used as a bridging strategy prior to allo-HSCT.
C - Comparator	Salvage chemotherapy ± TKI, blinatumomab, inotuzumab ozogamicin, other eligible non-CAR-T approaches; historical controls accepted.
O - Outcomes	Primary: OS, LFS, DFS, EFS, PFS, GRFS. Secondary: CIR, NRM, pre-transplant MRD negativity, hematopoietic engraftment, acute/chronic GVHD, CMV/EBV reactivation, VOD, TA-TMA, CAR-T-specific toxicities.

Data Sources and Search Strategy

PubMed/MEDLINE and Scopus were searched from database inception to August 18, 2026. The search strategy was designed to maximize sensitivity and did not require the terms “bridge,” “bridging,” “relapsed/refractory,” or “MRD,” because eligible studies could describe the relevant treatment sequence without explicitly using these terms in the title or abstract. Both databases were searched using the same three conceptual domains: B-ALL/acute lymphoblastic leukemia, CAR-T/chimeric antigen receptor T-cell therapy, and allo-HSCT/allogeneic hematopoietic stem cell transplantation, with adaptations to the syntax and field structure of each database.

The exact PubMed/MEDLINE search strategy was: ((“acute lymphoblastic leukemia”[Title/Abstract] OR “acute lymphoblastic leukaemia”[Title/Abstract] OR “B-cell acute lymphoblastic leukemia”[Title/Abstract] OR “B-cell acute lymphoblastic leukaemia”[Title/Abstract] OR “B lymphoblastic leukemia”[Title/Abstract] OR “B lymphoblastic leukaemia”[Title/Abstract] OR “B-ALL”[Title/Abstract]) AND (“CAR-T”[Title/Abstract] OR “CAR T”[Title/Abstract] OR “CAR T-cell”[Title/Abstract] OR “CAR T cell”[Title/Abstract] OR “chimeric antigen receptor T”[Title/Abstract] OR “chimeric antigen receptor T-cell”[Title/Abstract]) AND (“allo-HSCT”[Title/Abstract] OR “allo-HCT”[Title/Abstract] OR “allogeneic hematopoietic stem cell transplantation”[Title/Abstract] OR “allogeneic haematopoietic stem cell transplantation”[Title/Abstract] OR “allogeneic hematopoietic cell transplantation”[Title/Abstract] OR “allogeneic haematopoietic cell transplantation”[Title/Abstract] OR “allogeneic stem cell transplantation”[Title/Abstract] OR “haplo-HSCT”[Title/Abstract] OR “haploidentical hematopoietic stem cell transplantation”[Title/Abstract] OR “haploidentical haematopoietic stem cell transplantation”[Title/Abstract])).

The equivalent Scopus search strategy was: TITLE-ABS-KEY(“acute lymphoblastic leukemia” OR “acute lymphoblastic leukaemia” OR “B-cell acute lymphoblastic leukemia” OR “B-cell acute lymphoblastic leukaemia” OR “B lymphoblastic leukemia” OR “B lymphoblastic leukaemia” OR “B-ALL”) AND TITLE-ABS-KEY(“CAR-T” OR “CAR T” OR “CAR T-cell” OR “CAR T cell” OR “chimeric antigen receptor T” OR “chimeric antigen receptor T-cell”) AND TITLE-ABS-KEY(“allo-HSCT” OR “allo-HCT” OR “allogeneic hematopoietic stem cell transplantation” OR “allogeneic haematopoietic stem cell transplantation” OR “allogeneic hematopoietic cell transplantation” OR “allogeneic haematopoietic cell transplantation” OR “allogeneic stem cell transplantation” OR “haplo-HSCT” OR “haploidentical hematopoietic stem cell transplantation” OR “haploidentical haematopoietic stem cell transplantation”).

The search retrieved 527 records from PubMed/MEDLINE and 1,430 records from Scopus.

Eligibility Criteria

Studies were eligible if they included patients with R/R B-ALL, or patients in CR1 with persistent/recurrent MRD, who received CAR-T therapy as a bridging strategy before allo-HSCT and were compared with a non-CAR-T bridging strategy as defined above. Eligible comparative studies were required to provide post-transplant outcomes that could be evaluated according to the pre-transplant bridging strategy received.

Studies were excluded when CAR-T was evaluated without a relevant non-CAR-T pre-transplant comparator; when the main comparison was transplantation versus no transplantation after CAR-T therapy; when CAR-T was administered after a prior allo-HSCT; when CAR-T and chemotherapy were compared only as salvage or reinduction strategies without a subsequent comparative bridging-to-transplant analysis; when different CAR-T constructs were compared without a non-CAR-T arm; or when post-transplant outcomes could not be stratified according to the pre-transplant strategy.

Only original research articles involving human participants and published in English or French were considered. This language restriction reflected the review team’s working languages and was considered a potential source of language bias.

Study Selection

Records retrieved from the database searches were imported into Rayyan (Qatar Computing Research Institute, Ar-Rayyan, Qatar) for duplicate removal and screening. Two reviewers independently screened titles and abstracts, and reports judged potentially eligible underwent full-text assessment against the predefined eligibility criteria. Disagreements at either stage were resolved through discussion until consensus was reached.

Data Extraction

Data were extracted independently by two reviewers using a standardized data collection form developed before the review began. Extracted variables included study characteristics, study design, patient demographics, disease status, sample size, CAR-T construct, comparator treatment, the proportion of CAR-T-treated patients who proceeded to allo-HSCT, remission and MRD status, survival outcomes as defined by each study, cumulative incidence of relapse (CIR), NRM, hematopoietic engraftment, GVHD, infectious complications, and other transplant-related toxicities. Discrepancies in data extraction were resolved through discussion.

Assessment of Methodological Quality

Methodological quality was independently assessed by two reviewers using the appropriate Joanna Briggs Institute (JBI) critical appraisal tool for each study design. Comparative cohort studies were evaluated using the JBI Critical Appraisal Checklist for Cohort Studies described by Moola et al. [12], while the single quasi-experimental study was assessed using the revised JBI Critical Appraisal Checklist for Quasi-Experimental Studies described by Barker et al. [13].

Each domain was rated as “Yes,” “No,” “Unclear,” or “Not applicable.” Items were rated as “Unclear” when the published report provided insufficient information to permit a reliable judgment, rather than being assumed favorable by default. Disagreements between the two reviewers were resolved through discussion until consensus was reached. These quality assessments informed the interpretation of individual studies within the narrative synthesis rather than serving as an exclusion criterion.

Data Synthesis

A meta-analysis was not performed because of substantial clinical and methodological heterogeneity across the included reports. Populations ranged from patients in CR1 with persistent/recurrent MRD to those with overt R/R disease; comparators included conventional chemotherapy, chemotherapy with a tyrosine kinase inhibitor (TKI), blinatumomab, and historical controls; CAR-T constructs varied between single-target CD19 and dual-target CD19/CD22 products; and outcome definitions varied across studies.

A narrative synthesis was performed instead, with outcomes grouped into disease response and MRD status, survival and relapse, NRM, hematopoietic engraftment, GVHD, infectious complications, and other toxicities. Where both adjusted and unadjusted estimates were available, adjusted hazard ratios or odds ratios with 95% confidence intervals were given priority, and isolated unadjusted p-values were interpreted cautiously.

All included reports provided analyzable post-transplant outcomes stratified by pre-transplant bridging strategy; however, in some studies, only a subset of the original treatment cohort proceeded to allo-HSCT. This was considered a potential source of transplant-eligibility and selection bias throughout the review.

Finally, one pair of included reports originated from the same institution and had overlapping recruitment periods, and fully independent patient cohorts could not be confirmed. This potential overlap was handled qualitatively without adjustment of the reported sample sizes. The findings of these two reports were therefore interpreted cautiously and were not considered mutually confirmatory.

Results

Study Selection

The literature search identified 1,957 records from PubMed/MEDLINE (n=527) and Scopus (n=1,430). After removal of 500 duplicates, 1,457 records remained for title and abstract screening, of which 1,391 were excluded. Sixty-six reports were sought for full-text retrieval and assessed for eligibility; 56 were excluded because they did not meet the predefined eligibility criteria.

The most common reasons for full-text exclusion were the absence of post-transplant outcomes stratified by pre-transplant bridging strategy, comparison of transplantation versus no transplantation after CAR-T therapy rather than comparison of bridging strategies, and non-comparative single-arm CAR-T-to-HSCT designs.

Ten reports met all eligibility criteria and were included in the narrative synthesis (Figure 1).

**Figure 1:**
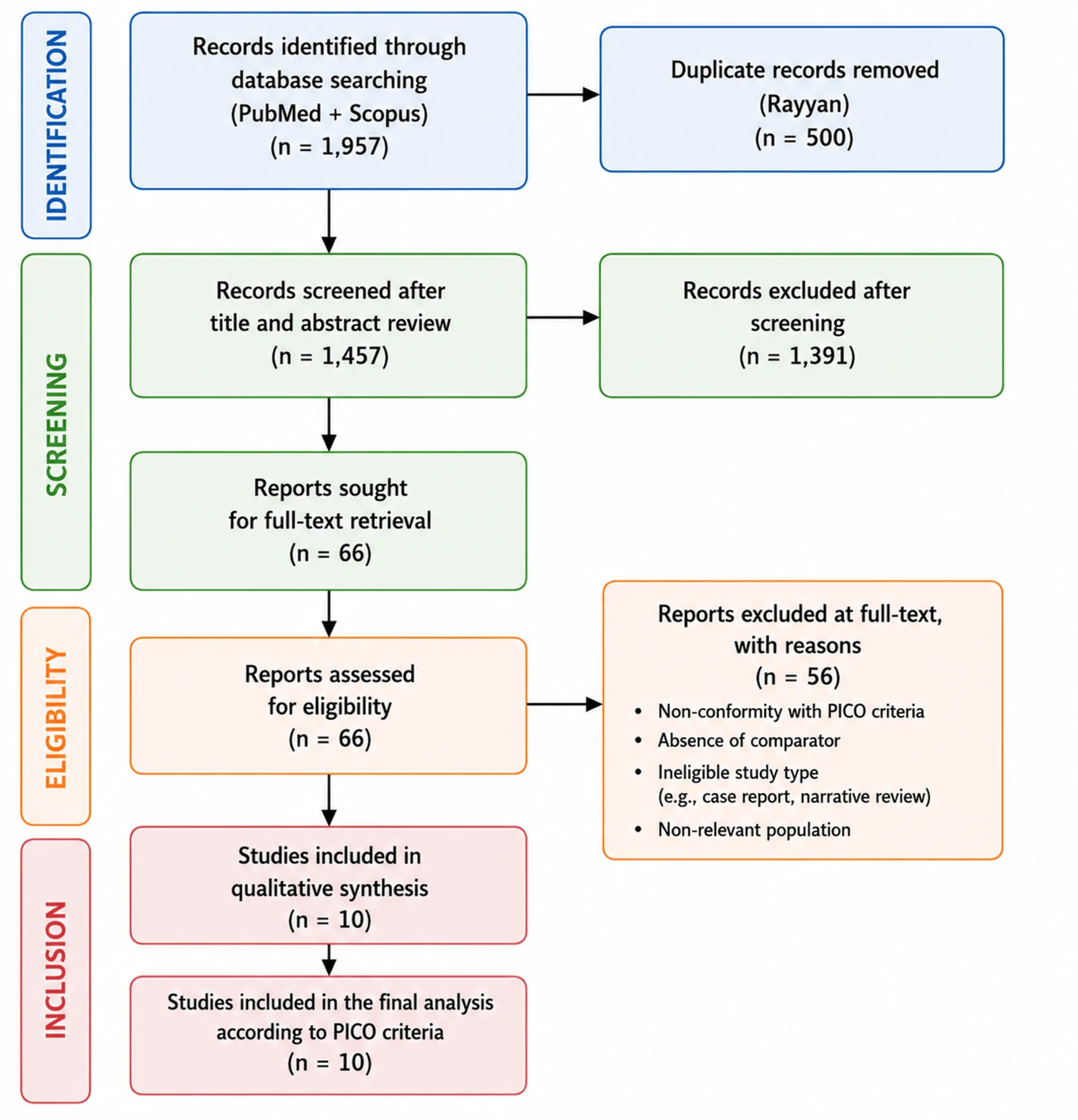
PRISMA 2020 Flow Diagram of the Study Selection Process Two of the included reports originated from the same institution and had overlapping recruitment periods; therefore, potential patient-level overlap between these reports could not be excluded.

Study Characteristics

The included reports were heterogeneous with respect to patient age, disease status before transplantation, CAR-T construct, transplant platform, and non-CAR-T comparator (Table 2).

**Table 2:** Characteristics of the Included Studies ^a ^Yao et al. [19]: 25 patients were enrolled in the prospective CAR-T trial; 21 achieved CR/CRi, and 17 proceeded to allo-HSCT. ^b^ The reported CAR-T groups in Yang et al. [20] and Zhao et al. [23] comprised patients who achieved remission and proceeded to transplantation; the percentage therefore refers to the analyzed cohort rather than all patients initially treated with CAR-T. allo-HSCT, allogeneic hematopoietic stem cell transplantation; B-ALL, B-cell acute lymphoblastic leukemia; CAR-T, chimeric antigen receptor T-cell; CR, complete remission; CR1, first complete remission; CR2, second complete remission; CRi, complete remission with incomplete hematologic recovery; haplo-HSCT, haploidentical hematopoietic stem cell transplantation; MRD, measurable residual disease; NR, not reported; Ph, Philadelphia chromosome; PSM, propensity score matching; R/R, relapsed/refractory; TKI, tyrosine kinase inhibitor

Characteristic	Wu et al. (2026) [14]	Cao et al. (2025) [15]	Hu et al. (2025) [16]	Li et al. (2023) [17]	Zhao et al. (2023) [18]	Yao et al. (2023) [19]	Yang et al. (2022) [20]	Hu et al. (2022) [21] (first relapse)	Hu et al. (2022) [22] (CR1 MRD+)	Zhao et al. (2021) [23]
Journal	Eur J Haematol	J Transl Med	Chin Med J	Clin Transl Med	Chin Med J	Bone Marrow Transplant	Front Immunol	Front Immunol	Front Immunol	Front Immunol
Design	Retrospective cohort (PSM)	Retrospective cohort	Retrospective cohort (letter)	Retrospective cohort, 3-arm (letter)	Retrospective cohort (letter)	Prospective CAR-T cohort with historical comparator	Retrospective cohort	Retrospective cohort	Prospective cohort	Retrospective cohort
Period	2018-2021	2017-2023	2021-2022	2017-2022	2016-2020	2017-2020	2016-2021	2015-2020	2015-2019	2015-2016
Population	R/R B-ALL + CR1 MRD+	R/R B-ALL	Pediatric Ph-negative B-ALL	B-ALL in CR	Adult/pediatric R/R B-ALL	Relapsed Ph+ B-ALL	MRD-negative CR pre-haplo-HSCT	Pediatric first relapse, MRD-guided	Pediatric Ph-negative MRD+ CR1	Adult/pediatric R/R B-ALL
Age	≥14 years	Mixed	<18 years	Mixed	<60 years	Adults	Adults	2-18 years	1-18 years	Mixed
Comparator	Chemo ± TKI	Blinatumomab	Blinatumomab	Non-CAR-T	Chemotherapy	Historical chemotherapy salvage	Chemo (CR1/≥CR2)	Chemotherapy	Chemotherapy	Chemotherapy
Analyzed population, n	443	36	26	169	82	40	168	40	77	105
CAR-T-treated, n	50	27	14	72	37	25^a^	28	30	43	27
CAR-T→allo-HSCT, n (%)	50 (100%)	27 (100%)	14 (100%)	72 (100%)	37 (100%)	17 (68%)	28 (100%)^b^	26 (86.7%)	35 (81.4%)	27 (100%)^b^
Median follow-up, months	30 (1-62)	28	14.6-15.3	NR	30 vs. 24	33 (19-50)	31	Not clearly reported	44 (18-70)	49

Most studies evaluated conventional chemotherapy-based bridging strategies, whereas Cao et al. [15] and Hu et al. [16] directly compared CAR-T therapy with blinatumomab. The proportion of CAR-T-treated patients who proceeded to allo-HSCT also varied across studies. In some reports, the analyzed CAR-T cohort was defined by successful progression to transplantation and therefore did not represent all patients initially treated with CAR-T.

A potential patient-level overlap could not be excluded between the first-relapse pediatric cohort reported by Hu et al. [21] and the broader R/R B-ALL cohort reported by Zhao et al. [18], as both originated from Peking University People’s Hospital and had overlapping recruitment periods. These two reports were therefore interpreted cautiously throughout the synthesis, and their sample sizes were not combined when describing the overall evidence base.

Disease Response and Pre-transplant MRD

Pre-transplant disease response and MRD status were reported heterogeneously across the included studies, and several cohorts enrolled patients only after complete remission had been achieved, limiting direct comparisons of response rates between bridging strategies. In Wu et al. [14], pre-transplant MRD negativity was nearly identical after CAR-T and chemotherapy bridging (86.0% vs. 85.8%; p = 0.962), whereas Zhao et al. [23] reported a numerically higher but not statistically significant rate after CAR-T (77.8% vs. 65.4%; p = 0.232). In the pediatric cohorts reported by Hu et al. [21,22], CAR-T therapy was associated with high rates of MRD negativity, including a significantly lower residual disease burden than chemotherapy in the first-relapse cohort (median MRD, 0.009% vs. 0.304%; p = 0.006). Similarly, Cao et al. [15] reported high pre-transplant MRD-negative rates with both CAR-T and blinatumomab (88.9% vs. 77.8%; p = 0.587). Other studies either reported MRD outcomes descriptively or included only patients who had already achieved MRD-negative remission before transplantation, limiting assessment of differential MRD clearance. Overall, CAR-T-based bridging was capable of producing deep pre-transplant remissions; however, the available comparative evidence did not demonstrate a consistent advantage over non-CAR-T strategies in achieving MRD negativity.

Clinical Outcomes

Clinical outcomes by treatment strategy are summarized in Table 3.

**Table 3:** Survival and Disease-Control Outcomes According to Bridging Strategy ^a^ Restricted to patients who actually proceeded to allo-HSCT. allo-HSCT, allogeneic hematopoietic stem cell transplantation; CAR-T, chimeric antigen receptor T-cell; CIR, cumulative incidence of relapse; CR1, first complete remission; DFS, disease-free survival; EFS, event-free survival; LFS, leukemia-free survival; MRD, measurable residual disease; NR, not reported; NS, not statistically significant; OS, overall survival; PFS, progression-free survival; y, year

Endpoint (CAR-T vs control)	Wu et al. (2026) [14]	Cao et al. (2025) [15]	Hu et al. (2025) [16]	Li et al. (2023) [17]	Zhao et al. (2023) [18]	Yao et al. (2023) [19]	Yang et al. (2022) [20]	Hu et al. (2022) [21] (1st relapse)	Hu et al. (2021) [22] (CR1 MRD+)	Zhao et al. (2021) [23]
OS	90.9% vs. 94.6%; p=0.158	73.89% vs. 88.89% (2-y); p=0.862	92.9% vs. 75.0% (1-y); p=0.213	1-y; p=0.91	2-y; p=0.17	58.8% (2-y CAR-T cohort); p=0.365	87.9% vs. 71.5% (2-y); p=0.24	84.6% vs. 40.0% (3-y); p=0.008	85.6% vs. 73.3% (3-y)^a^; p=0.382	70.2% vs. 65.4% (4-y); p=0.681
LFS/DFS/EFS/PFS	LFS 80.0% vs. 86.8%; p=0.040	PFS 59.03% vs. 44.44%; p=0.501	DFS 64.3% vs. 75.0%; p=0.615	1-y; p=0.40	DFS 82.5% vs. 53.7%; p=0.01	EFS 52.9%; p=0.425	LFS 72.0% vs. 66.8%; p=0.85	LFS 71.8% vs. 44.4%; p=0.19	LFS 75.0% vs. 68.7%^a^; p=0.586	LFS 70.2% vs. 64.1%; p=0.63
CIR	9.1% vs. 8.4%; p=0.513	NR separately	7.1% vs. 8.3%; p=0.182	p=0.16	18.9% vs. 42.2%; p=0.02	3/17 transplanted	24.1% vs. 19.4%; NS	26.9% vs. 50.0%	NR	11.1% vs. 12.8%; p=0.84

Overall Survival (OS)

OS did not differ significantly between CAR-T and non-CAR-T bridging in most studies. A significant unadjusted OS advantage was reported only in the small pediatric first-relapse cohort of Hu et al. [21] (three-year OS, 84.6% vs. 40.0%; p=0.008); however, potential patient-level overlap with the broader cohort reported by Zhao et al. [18] warrants cautious interpretation. In the largest cohort, Wu et al. [14] found no significant difference in unadjusted OS (90.9% vs. 94.6%; p=0.158), whereas multivariable analysis identified CAR-T bridging as independently associated with improved OS (HR=0.365; 95% CI, 0.154-0.857; p=0.025). This discordance between adjusted and unadjusted estimates highlights the potential influence of baseline differences and residual confounding. No significant OS differences were reported in the remaining comparative studies.

Leukemia-Free Survival (LFS), Disease-Free Survival (DFS), and Event-Free Survival (EFS)

Disease-control outcomes were heterogeneous across studies. Zhao et al. [18] reported significantly higher two-year DFS (82.5% vs. 53.7%; p=0.01) and lower CIR (18.9% vs. 42.2%; p=0.02) after CAR-T bridging, with CAR-T remaining independently associated with reduced relapse risk on multivariable analysis (HR=0.369; p=0.02). In contrast, Wu et al. [14] reported significantly lower LFS after CAR-T bridging (80.0% vs. 86.8%; p=0.040), despite similar relapse rates, suggesting that the difference was primarily related to NRM rather than disease recurrence. Yang et al. [20] found no overall LFS difference between bridging strategies, although patients receiving chemotherapy who underwent transplantation in second or later complete remission had worse LFS than CAR-T-bridged patients (HR=2.57; 95% CI, 1.041-6.343; p=0.041). The remaining studies did not demonstrate significant differences in LFS, DFS, EFS, or progression-free survival (PFS) between bridging strategies.

Non-relapse Mortality (NRM)

NRM showed no consistent difference between CAR-T and non-CAR-T bridging strategies. The main exception was the largest cohort, in which Wu et al. [14] reported higher NRM after CAR-T bridging (10.9% vs. 4.3%; p=0.016); after propensity-score matching, the numerical difference persisted but was no longer statistically significant (12.0% vs. 5.0%; p=0.100). Other comparative studies, including Zhao et al. [23], Yang et al. [20], Cao et al. [15], and Li et al. [17], did not demonstrate significant differences in NRM. Overall, the available evidence does not indicate a consistent increase in NRM after CAR-T-based bridging, although the signal observed in the largest cohort warrants consideration alongside other post-transplant complications.

Hematopoietic Engraftment

Most studies reported broadly comparable neutrophil recovery between bridging strategies, whereas delayed platelet recovery emerged as a more recurrent signal after CAR-T therapy. Wu et al. [14] reported delayed platelet recovery (17 vs. 14 days; p<0.001) and a higher rate of platelet engraftment failure (14.0% vs. 3.5%) after CAR-T bridging. Zhao et al. [23] similarly observed delayed platelet recovery after CAR-T (14 vs. 12 days; p=0.026). In Li et al. [17], 28-day platelet engraftment was lower after dual-target CD19/CD22 CAR-T than after non-CAR-T bridging (69.0% vs. 87.4%; p=0.008), and dual-target CAR-T remained independently associated with slower platelet recovery (OR=2.80; 95% CI, 1.03-7.30; p=0.030). Other studies did not identify meaningful differences in hematopoietic recovery. Thus, delayed platelet engraftment appears to be a potential, but not universal, safety signal, particularly in cohorts receiving dual-target CD19/CD22 CAR-T therapy.

Post-transplant Complications

Post-transplant complications by treatment strategy are summarized in Table 4.

**Table 4:** Post-transplant Safety Outcomes According to Bridging Strategy CAR-T, chimeric antigen receptor T-cell; CMV, cytomegalovirus; CR1, first complete remission; EBV, Epstein-Barr virus; GVHD, graft-versus-host disease; MRD, measurable residual disease; NR, not reported; NS, not statistically significant; TA-TMA, transplant-associated thrombotic microangiopathy; TMA, thrombotic microangiopathy; VOD, veno-occlusive disease

Outcome (CAR-T vs. control)	Wu et al. (2026) [14]	Cao et al. (2025) [15]	Hu et al. (2025) [16]	Li et al. (2023) [17]	Zhao et al. (2023) [18]	Yao et al. (2023) [19]	Yang et al. (2022) [20]	Hu et al. (2022) [21] (1st relapse)	Hu et al. (2021) (CR1 MRD+) [22]	Zhao et al. (2021) [23]
Acute GVHD	Grade II-IV: 34.5% vs. 30.9%; p=0.656	22.2% vs. 33.3%; p=0.660	28.5% vs. 33.3%; NS	p=0.33	21.6% vs. 51.1%; p=0.01	CAR-T: 29.4%; comparator NR	17.9% vs. 19.3%; p=0.842	8/26 vs. 3/14	24% vs. 23%; p=0.956	48.1% vs. 25.6%; p=0.016
Chronic GVHD	30.9% vs. 44.0%; p=0.020	14.8% vs. 22.2%; p=0.627	35.7% vs. 41.6%; NS	p=0.54	NR	6/14 evaluable CAR-T	38.6% vs. 32.1%; p=0.918	16/26 vs. 9/14	56% vs. 49%; p=0.687	73.3% vs. 55.0%; p=0.107
CMV/EBV	CMV: 74% vs. 77%; EBV: 12% vs. 16.7%	CMV: 66.6% vs. 44.4%; NS	CMV: 57.1% vs. 75.0%; EBV: 7.1% vs. 16.7%	Dual CAR-T CMV: 65.5% vs. 28.6%; EBV, any CAR-T: 15.4% vs. 2.1%	Viral infection: 67.6% vs. 75.6%; p=0.42	3 infection-related deaths	CMV: 75.5% vs. 67.2%; EBV: 58.4% vs. 46.3%	CMV: 73.1% vs. 65.0%; EBV: 11.5% vs. 14.2%	NR	CMV: 52% vs. 50%; p=0.93
Endothelial complications	VOD: 4.0% vs. 0.5%; p=0.003	TMA: 11.1% vs. 11.1%	NR	TMA: p=0.56	NR	1 GVHD-related TMA	NR	TA-TMA: 1/26 vs. 0/14	NR	TMA: 15% vs. 14%; p=0.51

Graft-Versus-Host Disease (GVHD)

GVHD outcomes were heterogeneous across studies. Wu et al. [14] reported significantly lower chronic GVHD after CAR-T bridging (30.9% vs. 44.0%; p=0.020), with similar rates of grade II-IV acute GVHD, while Zhao et al. [18] observed significantly lower grade II-IV acute GVHD after CAR-T (21.6% vs. 51.1%; p=0.01). In contrast, Zhao et al. [23] reported higher grade II-IV acute GVHD after CAR-T (48.1% vs. 25.6%; p=0.016), although rates of grade III-IV acute GVHD were similar between groups. The remaining comparative studies did not demonstrate significant differences in GVHD. Overall, the available evidence does not indicate a consistent increase or reduction in GVHD after CAR-T-based bridging.

Infectious Complications

The most notable infectious signal was reported by Li et al. [17], in whom cytomegalovirus (CMV) viremia was more frequent after dual-target CD19/CD22 CAR-T than after non-CAR-T bridging (65.5% vs. 28.6%; p<0.001). Epstein-Barr virus (EBV) viremia was also more frequent when the CAR-T groups were combined (15.4% vs. 2.1%; p=0.001), and prior CAR-T exposure remained independently associated with EBV viremia (HR=8.52; 95% CI, 1.48-49.1; p=0.017). Other comparative studies did not identify significant differences in viral reactivation between bridging strategies. Thus, increased CMV and EBV reactivation appears to represent a potential safety signal in specific cohorts, particularly following dual-target CD19/CD22 CAR-T therapy, rather than a consistent finding across all CAR-T-based bridging strategies.

Veno-Occlusive Disease (VOD) and Thrombotic Microangiopathy

Wu et al. [14] reported a higher incidence of VOD after CAR-T than after chemotherapy bridging (4.0% vs. 0.5%; p=0.003), although only two VOD events occurred in the CAR-T group and the difference was no longer statistically significant after propensity-score matching. Other studies did not demonstrate a significant increase in transplant-associated thrombotic microangiopathy (TA-TMA) after CAR-T-based bridging, while Yao et al. [19] reported a single GVHD-associated TMA event in the CAR-T cohort. Overall, the available evidence does not demonstrate a consistent increase in endothelial complications after CAR-T-based bridging.

Risk of Bias and Methodological Quality

Nine reports were assessed using the JBI Critical Appraisal Checklist for Cohort Studies [12], while Yao et al. [19] was assessed using the JBI Critical Appraisal Checklist for Quasi-Experimental Studies [13]. The results of the methodological quality assessment are summarized in Table 5.

**Table 5:** JBI Methodological Quality Appraisal CR1, first complete remission; JBI, Joanna Briggs Institute; MRD, measurable residual disease

Study	Tool	Score	Principal domains rated, No/Unclear
Wu et al. (2026) [14]	Cohort	9/11	Follow-up completeness and strategy for losses insufficiently described
Cao et al. (2025) [15]	Cohort	9/11	MRD reporting inconsistency; follow-up completeness unclear
Hu et al. (2025) [16]	Cohort	6/11	Confounding adjustment and several reporting domains limited by letter format
Li et al. (2023) [17]	Cohort	6/11	Baseline comparability, follow-up, and confounding strategy incompletely reported
Zhao et al. (2023) [18]	Cohort	7/11	Exposure assignment, confounding, and follow-up incompletely reported
Yao et al. (2023) [19]	Quasi-experimental	6/9	Historical comparator and comparability of co-interventions/follow-up
Yang et al. (2022) [20]	Cohort	10/11	Strategy for incomplete follow-up not explicit
Hu et al. (2022) [21] (first relapse)	Cohort	9/11	Baseline MRD imbalance; follow-up completeness unclear
Hu et al. (2021) [22] (CR1 MRD+)	Cohort	10/11	Strategy for incomplete follow-up not explicit
Zhao et al. (2021) [23]	Cohort	8/11	Baseline imbalance and incomplete follow-up assessment

The methodological quality assessment was used to inform the interpretation of individual studies rather than to establish a formal ranking or exclusion threshold. The most favorable appraisal profiles were observed for Yang et al. [20] and Hu et al. [22], each meeting 10 of 11 JBI cohort criteria, followed by Wu et al. [14], Cao et al. [15], and Hu et al. [21], each meeting 9 of 11 criteria. Nevertheless, the evidence base remained predominantly non-randomized and therefore susceptible to residual confounding and selection bias.

Lower appraisal scores were observed in the reports by Hu et al. [16] and Li et al. [17], both of which met 6 of 11 cohort criteria, and Zhao et al. [18], which met 7 of 11. Several domains in these reports were rated as unclear because methodological information was insufficiently reported, particularly in publications presented as letters or correspondence. Findings derived primarily from these studies were therefore interpreted with greater caution in the narrative synthesis.

Yao et al. [19], which met six of nine applicable quasi-experimental criteria, had an additional risk of bias related to the use of a non-contemporaneous historical comparator. Across the evidence base, the main recurring methodological concerns were selection into the transplanted population, baseline imbalances in disease status or MRD burden, residual confounding, and incomplete reporting of follow-up.

Discussion

This systematic review compared CAR-T-based with non-CAR-T bridging strategies before allo-HSCT in patients with R/R B-ALL or persistent/recurrent MRD during CR1. Anti-CD19 CAR-T therapy has substantially changed the therapeutic landscape of R/R B-ALL. In the pivotal ELIANA trial, Maude et al. [24] reported complete remission or complete remission with incomplete hematologic recovery in 81% of treated children and young adults, with MRD negativity in all responders. However, durable disease control is not universal. Limited CAR-T-cell persistence, CD19 antigen loss or downregulation, and other immune-escape mechanisms remain important causes of relapse, as reviewed by Xu et al. [9]. These mechanisms provide a biological and clinical rationale for consolidative allo-HSCT after CAR-T-induced remission in selected high-risk patients.

The present review included ten comparative reports [14-23], all originating from single-center institutions in China. Considerable heterogeneity was observed in patient age, disease status, cytogenetic profile, CAR-T construct, comparator strategy, and transplant platform. In addition, the proportion of CAR-T-treated patients who proceeded to transplantation varied across studies, and several reports defined the analyzed CAR-T cohort only after remission and successful progression to allo-HSCT. These differences introduce important transplant-eligibility and selection biases that should be considered when interpreting comparisons across studies.

CAR-T-based bridging was capable of inducing deep remissions before transplantation, but the comparative evidence did not demonstrate a consistent advantage over non-CAR-T strategies in achieving MRD negativity. Wu et al. [14] reported nearly identical pre-transplant MRD-negative rates after CAR-T and chemotherapy, while Zhao et al. [23] observed a numerical but nonsignificant difference. High MRD-negativity rates were also reported in pediatric cohorts, including that of Hu et al. [22]. These findings are consistent with the high molecular response rates reported in ELIANA [24] and earlier CAR-T studies such as Pan et al. [25], while emphasizing the importance of achieving deep remission before transplantation regardless of the bridging modality used.

Survival and disease-control outcomes were similarly heterogeneous. Zhao et al. [18] provided the clearest favorable disease-control signal, reporting higher DFS and lower CIR after CAR-T bridging, with CAR-T remaining associated with reduced relapse risk after multivariable adjustment. However, most included studies did not demonstrate a significant OS advantage. In the largest cohort, Wu et al. [14] reported lower unadjusted LFS and higher NRM after CAR-T despite similar relapse rates, whereas multivariable analysis identified CAR-T bridging as independently associated with improved OS. This discrepancy highlights the potential influence of baseline imbalances and residual confounding in observational comparisons.

Hu et al. [21] reported a significant unadjusted three-year OS advantage with CAR-T compared with chemotherapy. However, this finding should be interpreted cautiously because of the small sample size and the possibility of patient-level overlap with the cohort reported by Zhao et al. [18]. Furthermore, several other studies, including the direct CAR-T-versus-blinatumomab comparisons by Cao et al. [15] and Hu et al. [16], found no significant OS difference. Collectively, the available evidence therefore does not establish a consistent survival advantage of CAR-T over other bridging strategies.

Non-comparative and longer-term studies provide additional context. Pan et al. [25] demonstrated that CD19-directed CAR-T therapy can induce deep responses in heavily pretreated R/R B-ALL, while Yang et al. [26] reported durable outcomes after sequential CAR-T therapy and allo-HSCT. Jiang et al. [27] found that consolidative allo-HSCT after CAR-T-induced MRD-negative remission improved EFS and relapse-free survival (RFS) in higher-risk subgroups, although this did not translate into an OS advantage. Although these studies cannot establish comparative superiority over alternative bridging strategies, they support the biological and clinical rationale for sequential CAR-T therapy and allo-HSCT in selected patients.

Safety outcomes were also heterogeneous. Delayed platelet recovery emerged as one of the more recurrent post-transplant signals. Wu et al. [14] and Zhao et al. [23] reported delayed platelet engraftment after CAR-T bridging, while Li et al. [17] identified impaired platelet recovery particularly after dual-target CD19/CD22 CAR-T therapy. These findings are consistent with the propensity-score-matched analysis by Yang et al. [28], which reported a higher risk of platelet engraftment failure after allo-HSCT following CAR-T compared with chemotherapy. Nevertheless, this association was not consistently observed across all cohorts.

The mechanisms underlying delayed platelet recovery remain uncertain. Potential contributing factors include greater prior treatment exposure, prolonged CAR-T-associated cytopenias, inflammatory injury to the bone marrow microenvironment, disease burden, and differences in conditioning regimens. The stronger association reported with dual-target CAR-T by Li et al. [17] also raises the possibility of construct-specific or cumulative immune-mediated effects on subsequent hematopoietic recovery.

Infectious complications varied considerably across studies. Li et al. [17] reported higher CMV viremia after dual-target CD19/CD22 CAR-T and higher EBV viremia after prior CAR-T exposure. In contrast, Wu et al. [14], Zhao et al. [18], Zhao et al. [23], Hu et al. [21], Hu et al. [16], and Cao et al. [15] reported broadly comparable viral reactivation rates between bridging strategies. Increased CMV or EBV reactivation should therefore be regarded as a potential cohort- or construct-specific safety signal rather than a consistent consequence of CAR-T-based bridging.

NRM likewise showed no consistent increase after CAR-T bridging. Wu et al. [14] reported significantly higher NRM after CAR-T, with infections accounting for a substantial proportion of non-relapse deaths, although the difference was attenuated after propensity-score matching. Most other studies reported comparable NRM or treatment-related mortality between groups. This competing risk may partly explain why improved disease control in some cohorts did not consistently translate into improved OS.

Endothelial toxicity remains another potential but unconfirmed safety concern. Wu et al. [14] reported a higher incidence of hepatic VOD after CAR-T bridging, although only two events occurred in the CAR-T group and the association was no longer statistically significant after propensity-score matching. No other included study reproduced this finding. Similarly, TA-TMA did not show a consistent increase across the available comparative evidence.

Prior CAR-T exposure was not associated with a consistent GVHD pattern. Wu et al. [14] and Zhao et al. [18] reported lower rates of selected GVHD outcomes after CAR-T, whereas Zhao et al. [23] reported higher grade II-IV acute GVHD. The remaining comparative studies were largely neutral [15-17,19-22]. These heterogeneous findings suggest that transplant-specific factors, including donor type, graft source, conditioning regimen, and GVHD prophylaxis, may substantially influence GVHD risk independently of the preceding bridging strategy.

The increasing availability of alternative immunotherapeutic bridging strategies further complicates treatment selection. Cao et al. [15] and Hu et al. [16] directly compared CAR-T with blinatumomab and found no clear advantage for either strategy across survival, relapse, engraftment, GVHD, or treatment-related mortality. Although these small retrospective cohorts cannot establish therapeutic equivalence, they illustrate the transition from comparisons of CAR-T with conventional chemotherapy toward comparisons among several active immunotherapeutic strategies. The efficacy demonstrated for blinatumomab in the TOWER trial [6], and inotuzumab ozogamicin in the INO-VATE trial [7] further supports the need for direct comparative studies of contemporary bridging options.

Selection of a bridging strategy should therefore account for patient age, disease burden, prior CD19-directed therapy, antigen expression, cytogenetic and molecular risk, treatment availability, CAR-T manufacturing time, donor availability, and transplant eligibility. An important consideration is whether an alternative strategy can achieve sufficiently deep disease control without adding CAR-T-related toxicity before transplantation.

MRD assessment is central to this individualized approach. Monitoring after CD19-directed CAR-T therapy may be complicated by CD19 loss or downregulation, which can limit CD19-dependent detection methods [9]. Wang et al. [29] reported long-term pediatric data demonstrating high rates of MRD clearance and durable outcomes after CD19 CAR-T therapy in patients with R/R B-ALL or pre-treatment MRD re-emergence, while Chen et al. [30] proposed cytoplasmic CD79a as an alternative marker for post-CAR-T disease monitoring. Exploratory biomarkers may further refine patient selection for transplantation after CAR-T. Liu et al. [31] reported that higher post-CAR-T levels of IL-6, IL-10, and IFN-γ were associated with poorer OS among patients subsequently undergoing allo-HSCT; however, these findings remain exploratory and require prospective validation.

Limitations

Several limitations should be considered. First, all 10 included reports originated from single-center institutions in China, limiting the generalizability of the findings to other healthcare systems, CAR-T manufacturing platforms, transplant infrastructures, and patient populations.

Second, the evidence base was predominantly retrospective and non-randomized. Even studies using propensity-score matching or multivariable adjustment remain susceptible to residual and unmeasured confounding. Yao et al. [19] evaluated a prospective CAR-T cohort against a non-contemporaneous historical comparator, introducing additional uncertainty related to differences between treatment eras.

Third, substantial clinical and methodological heterogeneity existed in patient age, disease status, cytogenetic risk, CAR-T construct and target, comparator treatment, conditioning regimen, donor type, timing of transplantation, outcome definitions, and follow-up duration. The dual-target CD19/CD22 strategy evaluated by Li et al. [17], for example, was associated with delayed platelet recovery and increased viral reactivation. This heterogeneity precluded a meaningful quantitative meta-analysis.

Fourth, selection into the transplanted population differed substantially across reports. Several studies analyzed only patients who achieved remission and successfully proceeded to allo-HSCT, thereby excluding patients with treatment failure, severe toxicity, early relapse, or other events preventing transplantation. Such conditioning on successful progression to transplantation may preferentially select favorable responders and consequently overestimate the effectiveness of a bridging strategy when evaluated from treatment initiation.

Fifth, three reports - Hu et al. [16], Li et al. [17], and Zhao et al. [18] - were published as letters or correspondence with limited methodological detail. Consequently, several JBI domains were rated as unclear. These ratings primarily reflect insufficient reporting rather than demonstrated methodological deficiencies, and findings from these studies were therefore interpreted with additional caution.

Sixth, patient-level overlap could not be excluded between the first-relapse pediatric cohort reported by Hu et al. [21] and the broader R/R B-ALL cohort reported by Zhao et al. [18], both originating from Peking University People’s Hospital during overlapping recruitment periods. These reports were therefore interpreted cautiously, and their sample sizes were not treated as additive.

Finally, this systematic review was not prospectively registered, and eligibility was restricted to English- and French-language publications. Relevant studies published in other languages, particularly Chinese, may therefore have been missed. In addition, the full texts of some potentially relevant reports could not be obtained despite attempts to contact the corresponding authors through their institutional email addresses; no responses were received. These reports could therefore not be formally assessed for eligibility. Despite the search being updated through August 18, 2026, the absence of randomized trials and prospective multicenter comparisons remains a major limitation of the available evidence.

Clinical implications and future research

Taken together, the findings support an individualized approach to selecting bridging therapy before allo-HSCT in B-ALL, based on MRD status, disease biology, CAR-T construct, depth of remission, prior therapy, and transplant eligibility rather than a uniform preference for CAR-T or non-CAR-T strategies.

Prospective multicenter studies are needed to directly compare CAR-T with contemporary alternatives such as blinatumomab and inotuzumab ozogamicin, using standardized definitions of MRD, relapse, NRM, engraftment, GVHD, and infectious complications. Future studies should also determine whether construct-specific safety signals, particularly delayed platelet recovery and viral reactivation after dual-target CAR-T [17], are reproducible. They should also help identify which patients derive sufficient additional benefit from allo-HSCT after CAR-T-induced MRD-negative remission to justify the associated transplant-related risks.

## Conclusions

This systematic review of 10 comparative reports indicates that CAR-T-based bridging before allo-HSCT in B-ALL can produce deep pre-transplant remissions and, in selected cohorts, reduce post-transplant relapse, but does not consistently improve OS compared with non-CAR-T bridging strategies. Safety outcomes, including hematopoietic engraftment, GVHD, infectious complications, and endothelial toxicity, were heterogeneous across studies, with delayed platelet recovery and viral reactivation emerging as potential safety signals in specific cohorts. The discordance between adjusted and unadjusted survival estimates in the largest cohort, together with recurrent transplant-eligibility and selection biases across studies, highlights the potential influence of confounding in this predominantly retrospective, non-randomized, single-center evidence base.

These findings do not support a systematic preference for either CAR-T or non-CAR-T bridging. The choice of bridging strategy before allo-HSCT should instead be individualized according to MRD status, disease biology, CAR-T construct, the depth of remission achievable with alternative therapies, and transplant eligibility. Prospective multicenter studies directly comparing CAR-T with contemporary alternatives such as blinatumomab and inotuzumab ozogamicin, using standardized post-transplant outcome definitions and appropriate adjustment for baseline differences, are needed to identify which patients are most likely to benefit from CAR-T-based bridging.
